# Deformed One-Dimensional Covalent Organic Polymers for Enhanced CO Electroreduction to Methanol

**DOI:** 10.1021/acsnano.5c06511

**Published:** 2025-06-18

**Authors:** Yong Liu, Honglei Wang, Yun Song, Charles B. Musgrave III, Pei Xiong, Jiangtong Li, Geng Li, Libei Huang, Jianjun Su, Yinger Xin, Qiang Zhang, Weihua Guo, Mingming He, Tanglue Feng, Xing Li, Molly Meng-Jung Li, Peter A. van Aken, Hongguang Wang, William A. Goddard III, Ruquan Ye

**Affiliations:** † Department of Chemistry, State Key Laboratory of Marine Pollution, 53025 City University of Hong Kong, Hong Kong 999077, China; ‡ Chair Materials for Electrical Engineering and Electronics, Institute of Materials Science and Engineering and Institute of Micro and Nanotechnologies MacroNano, 26559 TU Ilmenau, Gustav-Kirchhoff-Str. 5, Ilmenau 98693, Germany; § Materials and Process Simulation Center, 6469 California Institute of Technology, Pasadena 91125, California, United States; ∥ Department of Applied Physics, 26680 Hong Kong Polytechnic University, Hong Kong 999077, China; ⊥ Division of Science, Engineering and Health Study, School of Professional Education and Executive Development, The Hong Kong Polytechnic University (PolyU SPEED), Hong Kong 999077, China; # 28326 Max Planck Institute for Solid State Research, Heisenbergstr. 1, Stuttgart 70569, Germany

**Keywords:** CO reduction, methanol, covalent organic polymer, strain, curvature

## Abstract

Spatially arranged molecular catalysts in polymeric frameworks, typically in a layered structure, are emerging strategies to mitigate the molecular aggregation and improve the catalytic performance. However, the effect of local coordination induced by polymerization remains underexplored. Here, we develop one-dimensional cobalt-tetra-amino-phthalocyanine-based covalent organic polymers (1D-COP) for the electrochemical CO reduction reaction (CORR). We use carbon nanotubes as ideal templates to induce local curvature of the 1D-COP. The COP on single-walled CNT (1D-COP/SWCNT) catalyst exhibits a maximum methanol Faradaic efficiency of 70% in an H-cell, which exceeds those on wider-diameter multiwalled carbon nanotubes (22% for 4–6 nm and 14% for 10–20 nm). Using X-ray and vibronic spectroscopies, we have observed distinct local geometries and electronic structures induced by the strong interactions between the COP layer and CNT substrates. Density functional theory calculations further support that increased curvature of the COP-SWCNT catalyst enhances the *CO binding species, leading to improved subsequent reduction reactions. Our results highlight the critical role of the local structure in polymeric frameworks for improved electrocatalytic performance.

## Graphical abstract

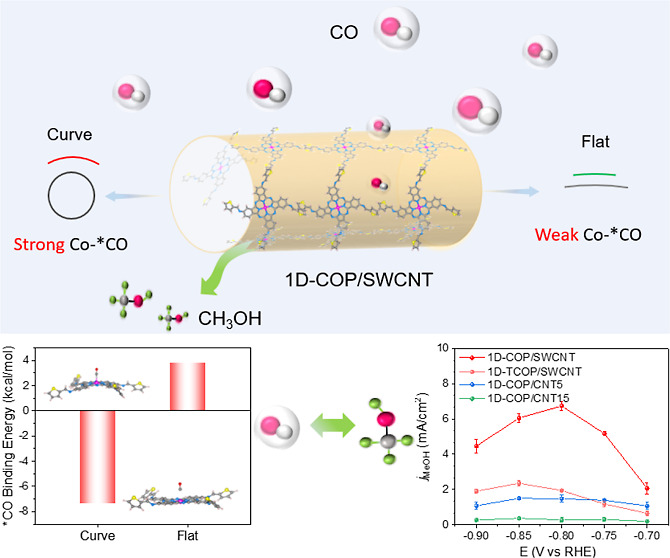

## Introduction

The electrocatalytic CO/CO_2_ reduction reaction (CO/CO_2_RR) offers a promising solution to address environmental concerns and produce valuable products.^1–4^ Among various products, methanol (CH_3_OH) is a promising target because it is not only a crucial intermediate for various chemical products^5,6^ but also a sustainable energy carrier.^7^ Currently, Cu-related catalysts are the most popular candidates for multielectron CO/CO_2_RR, which often produce a mixture of products that require further separation efforts.^8–10^ Molecular catalysts with well-defined structures can be modified by different functional groups to change the catalytic site of the molecule.^11–15^ Previous work has shown that cobalt phthalocyanine (CoPc) and cobalt-tetra-amino-phthalocyanine (CoTAPc) have the potential to produce methanol from CO/CO_2_RR.^16–21^ In 2019, it was demonstrated that high CO_2_-to-CH_3_OH selectivity can be achieved by immobilizing CoPc on multiwalled carbon nanotubes (MWCNTs).^22^ The crucial intermediate (*CO) with a high local concentration near catalytic sites is beneficial to improve methanol production.^23,24^ Similarly, single-walled carbon nanotubes (SWCNTs) with higher curvature induced different molecule–substrate interactions, resulting in a highly deformed CoPc.^25^ This interaction favored the adsorption of the *CO intermediate for subsequent reduction, ultimately resulting in a methanol Faradaic efficiency (FE_MeOH_) of 53.4%. These experimental results highlight the importance of uniformly dispersed CoPc molecules and the necessity of *CO for methanol production. Since the CO_2_-to-CO conversion has been improved to nearly 100% FE and *CO is considered to be the key reaction intermediate in methanol production, an ideal CORR catalyst could benefit tandem catalysis. Liu realized a CO-to-methanol Faraday efficiency of 65% by electron rearrangement of cobalt from a low-spin to a high-spin state.^26^ In addition, tuning the microenvironments in the electrical double layer also promotes methanol production.^27,28^

Molecular catalysts are typically physically mixed with carbon supports through π–π interaction, which is prone to aggregation, especially under high molecular loading.^29–32^ In recent years, researchers have proposed metal organic frameworks, hydrogen-bonded organic frameworks, covalent organic frameworks (COFs), and polymers (COPs) to address the aggregation problem by spatially arranging molecules.^33–37^ COFs and COPs, formed via condensation reactions of organic monomers, provide well-defined structures, high stability, and inherent porosity. However, COFs and COPs typically tend to form thick layers, which have lower electron conductivity and limit their multielectron efficiency.^38,39^ To improve the performance, COPs can be constructed in an ultrathin layered form, which are further mixed with carbon supports to enhance electron transfer.^40^ In this study, we demonstrate that in addition to improved molecular dispersion, COPs can induce changes in local coordination through template-directed polymerization, leading to tailored CORR activity. Specifically, one-dimensional (1D) ultrathin CoPc-based COPs with atomically isolated cobalt sites were synthesized by using SWCNT as a scaffold, denoted as 1D-COP/SWCNT. For comparison, 1D-COP on MWCNT (denoted as 1D-COP/CNTX, where X is the average diameter of the CNT in nm) and thick 1D-COP layers on SWCNT (1D-TCOP/SWCNT) were also synthesized, as evidenced by scanning transmission electron microscopy (STEM) studies. We found that 1D-COP/SWCNT exhibited the best CH_3_OH production with a maximum FE_MeOH_ of 70% at −0.8 V vs reversible hydrogen electrode (RHE; all the potentials are referenced to RHE unless otherwise noted). This efficiency exceeds that of 1D-COP/CNT5 (34%), 1D-COP/CNT15 (28%), and 1D-TCOP/SWCNT (36%). *In situ* Fourier transform infrared (FTIR) spectroscopic, Raman, X-ray spectroscopy, and density functional theory (DFT) calculations support the advantages of SWCNT template-directed 1D CoTAPc-based COPs, in which the higher curvature of SWCNT induces a greater degree of COP deformation. The local deformation, in turn, enhances the adsorption binding of *CO near the Co sites, consequently resulting in a higher CO-to-methanol conversion efficiency.

## Results and Discussion

Figure 1a shows the synthesis of 1D-COP/SWCNT, which shows the acetic-acid-catalyzed condensation reaction between trieno­[3,2-*b*] thiophene-2,5-dicarbaldehyde (Ttp) and CoTAPc on the surface of carbon supports. We use Ttp rather than the typical terephthalaldehyde to expand the linker library for constructing COPs. The ultrathin COP layer was deposited on the CNT surface *in situ* by template-oriented polymerization. In addition, the CNTs serve to adjust the morphology of COP and form ultrathin layers to disperse Co sites. For comparison, the control catalysts, including 1D-TCOP/SWCNT, 1D-COP/CNT5, 1D-COP/CNT15, and COP, were also prepared (please see the Supporting Information for detailed synthesis procedures). Since Ttp is used for synthesizing all of the COPs, the variation in the catalytic performance can be attributed to the support effect rather than the linker effect. The microstructures of these catalysts were first revealed by scanning electron microscopy (SEM) (Figure S1). The template-synthesized catalysts retain the tubular structures of the CNT, while pure COP forms agglomerate spherical shapes. Figures 1b,c and S2 compare low-angle annular dark-field scanning transmission electron microscopy (LAADF-STEM) images of 1D-COP/SWCNT, 1D-TCOP/SWCNT, 1D-COP/CNT5, and 1D-COP/CNT15, showing a uniform coating of 1D COP layer on the CNT surface. In addition, electron energy loss spectroscopy (EELS) elemental mapping confirms the uniform dispersion of Co, N, C, and S elements of COP on the CNT (Figure 1d). To further verify the successful preparation of COP on the CNT surface, the Frontier transform infrared (FT-IR) spectra of the materials were implemented. The intensity of N–H stretching peaks at 3316 and 3442 cm^–1^ from CoTAPc decreases or disappears in COPs (Figure S3). The C peaks (26° and 43°) instead of the metallic peaks of Co were observed in the X-ray diffraction (XRD) pattern (Figure S4), indicating that the CNTs were loaded with an amorphous COP film. The inductively coupled plasma optical emission spectroscopy (ICP-OES) analysis shows that the Co content in 1D-COP/SWCNT was 1.21 wt % (Table S1). When the COP was synthesized *in situ* and wound on the CNT surface, the characteristic peaks of COP and CNT were observed in the Raman spectra (Figures 2a and S5). Interestingly, 1D-COP/SWCNT not only shows the intrinsic peaks of 1D-COP and SWCNT but also exhibits a new peak at 221 cm^–1^ (Figures 2b and S5b). This peak originates from the out-of-plane deformation and ring-boating of Co–N, which is commonly found in double-walled CNTs.^25,41^ The peak intensities of 689 and 750 cm^–1^ are also different from the peaks of COP, 1D-COP/CNT5, and 1D-COP/CNT15 (Figure S5c–f). These signals suggest that there is a more pronounced interaction between the SWCNTs and the ultrathin COP film, resulting in changes in the vibrational peaks.

**Figure 1:**
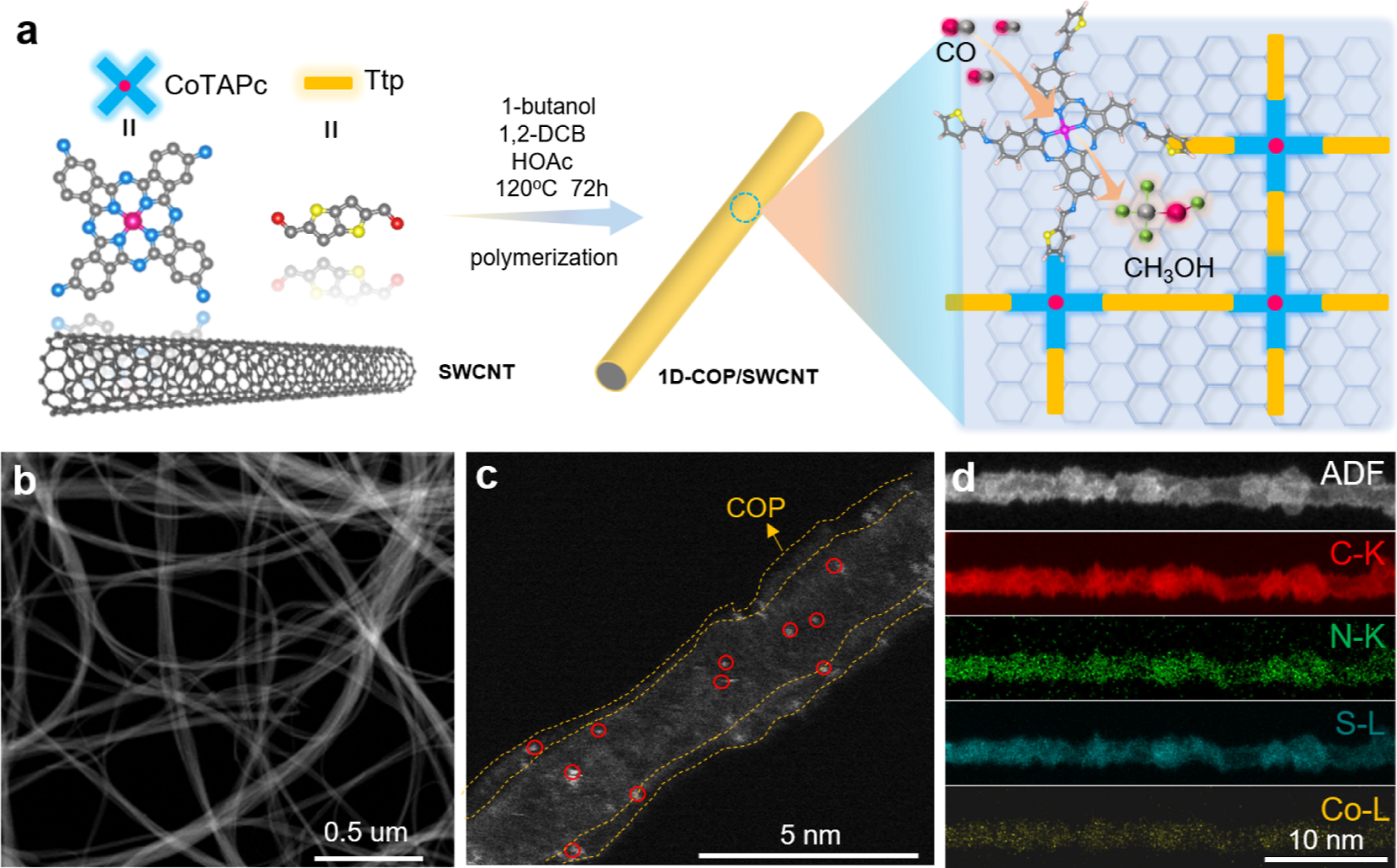
(a) Strategy and route for the synthesis of 1D-COP on SWCNTs. (b) Low-resolution STEM image. (c) Atomic-resolution LAADF-STEM image of 1D-COP on SWCNTs. The red circles highlight the dispersed Co atoms on the SWCNT surface. (d) EELS elemental mapping of 1D-COP/SWCNT in annular dark-field scanning (ADF) STEM image: C (red), N (green), S (light blue), Co (yellow).

**Figure 2:**
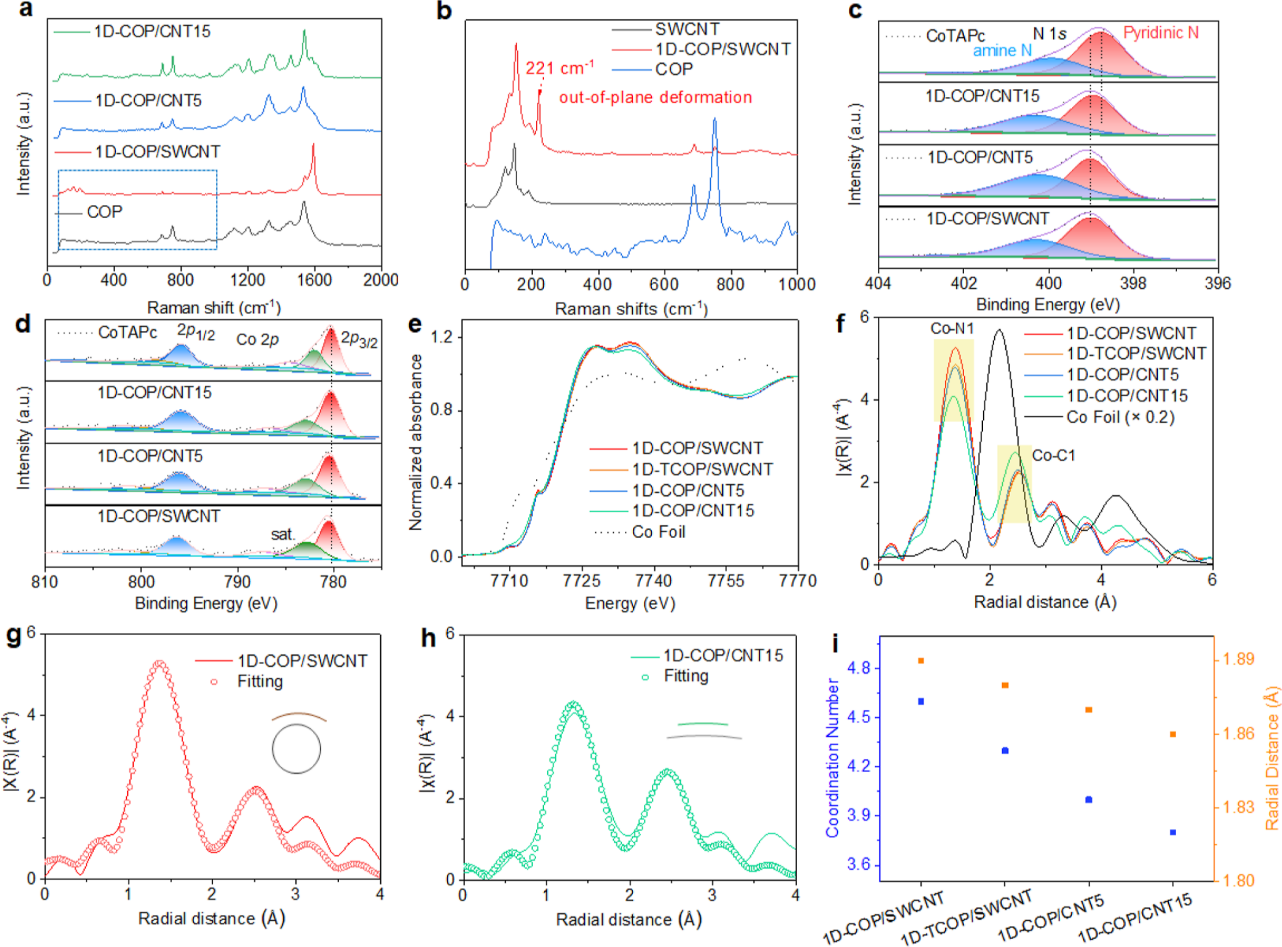
(a) Raman spectra and (b) enlarged area of COP, 1D-COP/SWCNT, 1D-TCOP/CNT5, 1D-COP/CNT15. (c) X-ray photoelectron spectroscopy (XPS) of N 1s. (d) Co 2p of CoTAPc, 1D-COP/SWCNT, 1D-TCOP/CNT5, 1D-COP/CNT15. (e) X-ray absorption near-edge structure (XANES) Co K-edge spectrum. (f) Fourier transform extended X-ray absorption fine structure (EXAFS). (g) Simulated structures and R-space EXAFS fit for 1D-COP/SWCNT and (h) 1D-COP/CNT15. The insets illustrate the local curvature in reference to molecule CoPc. (i) Coordination number and radial distance of 1D-COP/SWCNT, 1D-TCOP/SWCNT, 1D-TCOP/CNT5, 1D-COP/CNT15.

XPS was used to analyze the valence states of the four samples including CoTAPc and three different COP materials. The full XPS survey spectra show that they are all composed of C, N, S, O, and Co elements (Figure S6). Figure 2c,d shows the Co 2p1/2 and Co 2p3/2 peaks. Notably, the Co 2p1/2 and Co 2p3/2 peaks of 1D-COP/SWCNT show a shift toward higher binding energies. The N 1s spectra further accentuate the variation, where the nitrogen can be deconvoluted into two peaks at 399 and 400.4 eV. This observation is consistent with previous reports indicating that strong molecular–substrate interactions result in peak shifts and even peak splitting.^42,43^ Additionally, the reduction potential of Co shifts from −0.004 V (1D-COP/CNT15), −0.025 V (1D-COP/CNT5), −0.02 V (1D-TCOP/SWCNT) to −0.072 V (1D-COP/SWCNT) in Figure S7. Taken together, these results indicate the presence of distinct Co environments on CNT surfaces especially for 1D-COP/SWCNTs.^44^

In order to understand the local structural features of COP on different CNTs, XANES and EXAFS analyses were used to investigate the fine structure of the Co properties. The XANES (Figures 2e and S8) shows that there is a notable absorption in the K-edge of Co at 7715 eV, which is attributed to the 1s-4p transition in the Co–N4 structure.^45^ The peak near 7735 originates from the 1s-4*pxy* transition, which is closely related to the valence state of Co.^46^ The peak intensity decreases in the order of 1D-COP/SWCNT, 1D-COP/CNT5, and 1D-COP/CNT15, which correlates with the CNT structures.

Fourier-transform EXAFS mapping (Figure 2f) and EXAFS fitting in the R and *k* space are summarized in Figures 2g,h, S9, and S10, which show that the distances between Co–N1 and Co–C1 become shorter as the diameter of the CNT increases (Figure 2i and Table S2). Both Co–N1 and Co–C1 reflect the degree of deformation of the CoTAPc unit in COP.^25,47^ The peak intensity of the first coordination shell (Co–N) gradually diminishes as the CNT diameter increases from SWCNT to CNT15 (Figure 2f). Notably, the pronounced curvature of SWCNTs in 1D-COP/SWCNT induces a more distinct local structural distortion compared to larger-diameter CNT5 and CNT15. The increasing coordination number of Co results from the interaction of the CoTAPc molecule with the substrate, CNT (Figure 2i). Therefore, 1D-COP/SWCNT has longer Co–N1 and Co–C1 bond lengths than other samples, and the peak intensities are stronger due to the more intense interactions between the Co site and SWCNTs. These are in agreement with the experimental results in Raman and XPS.

The electrocatalytic CORR performance was first evaluated using the H-cell setup with linear sweep voltammetry (LSV) measurements in a CO-saturated 0.5 M K_2_HPO_4_ electrolyte. Among the different samples tested, 1D-COP/SWCNT exhibits a higher current density compared to the others (Figure 3a). The CORR selectivity of the four samples at different voltages was evaluated by chronoamperometric tests (Figure S11). Gas and liquid products were detected by using gas chromatography (GC) and ^1^H nuclear magnetic resonance spectroscopy (NMR; Figure S12), respectively. The primary products are CH_3_OH and H_2_, and the FE_MeOH_ shows a volcano-shaped trend as the voltage increases from −0.7 V to −0.9 V without *iR* compensation. The methanol selectivity of 1D-COP/SWCNT reaches a maximum of 70% at −0.8 V, along with a partial current density (j_MeOH_) of 6.7 mA/cm^2^, exceeding that of 1D-COP/CNT5 (22%, 1.4 mA/cm^2^), 1D-COP/CNT15 (14%, 0.3 mA/cm^2^), and COP (1.4%, 0.1 mA/cm^2^) (Figure 3b). These data indicate that the CNT diameter plays a critical role in the CO-to-CH_3_OH conversion Furthermore, the methanol selectivity of 1D-COP/SWCNT is also superior to that of 1D-TCOP/SWCNT (36%) (Figures 3c and S12), which is attributed to the weakened layer–support interactions. The high methanol selectivity achieved by ultrathin 1D-COP/SWCNTs exceeds the selectivity reported in several other studies (Figure 3d).^16,18,22,23,25,26^ These results highlight the potential of 1D-COP/SWCNT as a promising catalyst for the CO-to-CH_3_OH conversion.

**Figure 3:**
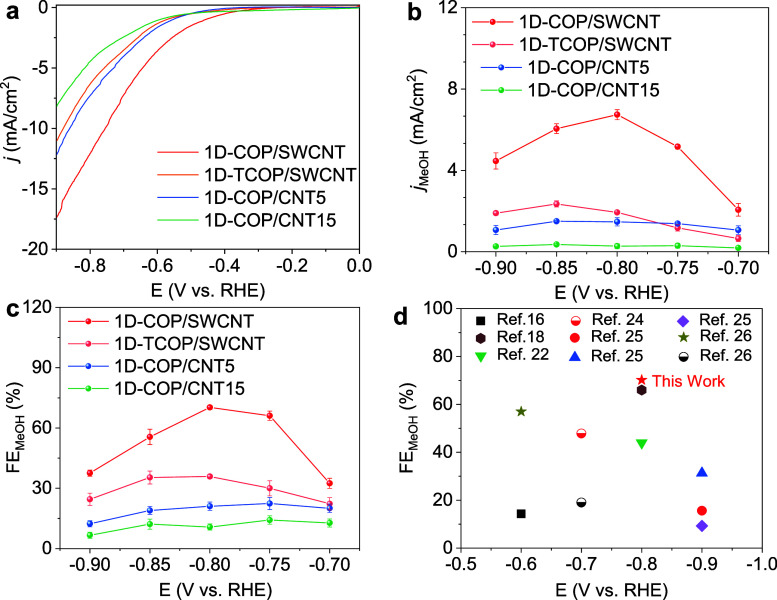
(a) LSV curves in CO-saturated 0.5 M K_2_HPO_4_ solution at a scan rate of 20 mV/s. The (b) current density, (c) product selectivity for CORR in H-cell with 0.5 M K_2_HPO_4_ solution as the electrolyte. (d) Comparison of the FE_MeOH_ for the 1D-COP/SWCNT with recently reported CoPc-based electrocatalysts.

The CORR was further evaluated in a flow-cell electrolyzer to mitigate the sluggish CO mass transport in an aqueous solution (Figure 4a). Here, we use 0.1 M KOH + 1.5 M KCl as the electrolyte because more alkaline conditions generally have higher inhibition of hydrogen evolution in flow cells.^12,31^ As shown in Figures 4b,S13, and S14, the products of 1D-COP/SWCNTs are hydrogen and methanol, which exhibit a total current density of 72 mA/cm^2^ with a methanol FE (FE_MeOH_) as high as 60% at −0.85 V. In comparison, 1D-TCOP/SWCNT, 1D-COP/CNT5, and 1D-COP/CNT15 exhibit lower FE_MeOH_ and *j*_MeOH_ (Figure 4c, d). Even though 1D-TCOP/SWCNT has nearly twice the cobalt (Co) content compared to other catalysts, many Co sites are covered by a thick layer and cannot interact with reactants for catalysis (Figures S7 and S15). Additionally, 1D-TCOP/SWCNT exhibits the highest charge transfer resistance among all samples (Figure S15f). As the CO-to-methanol process involves a proton-coupled electron transfer step, higher charge transfer resistance hinders the catalytic performance, particularly at high current in a flow cell. Consequently, 1D-TCOP/SWCNT demonstrates the poorest catalytic performance.

**Figure 4:**
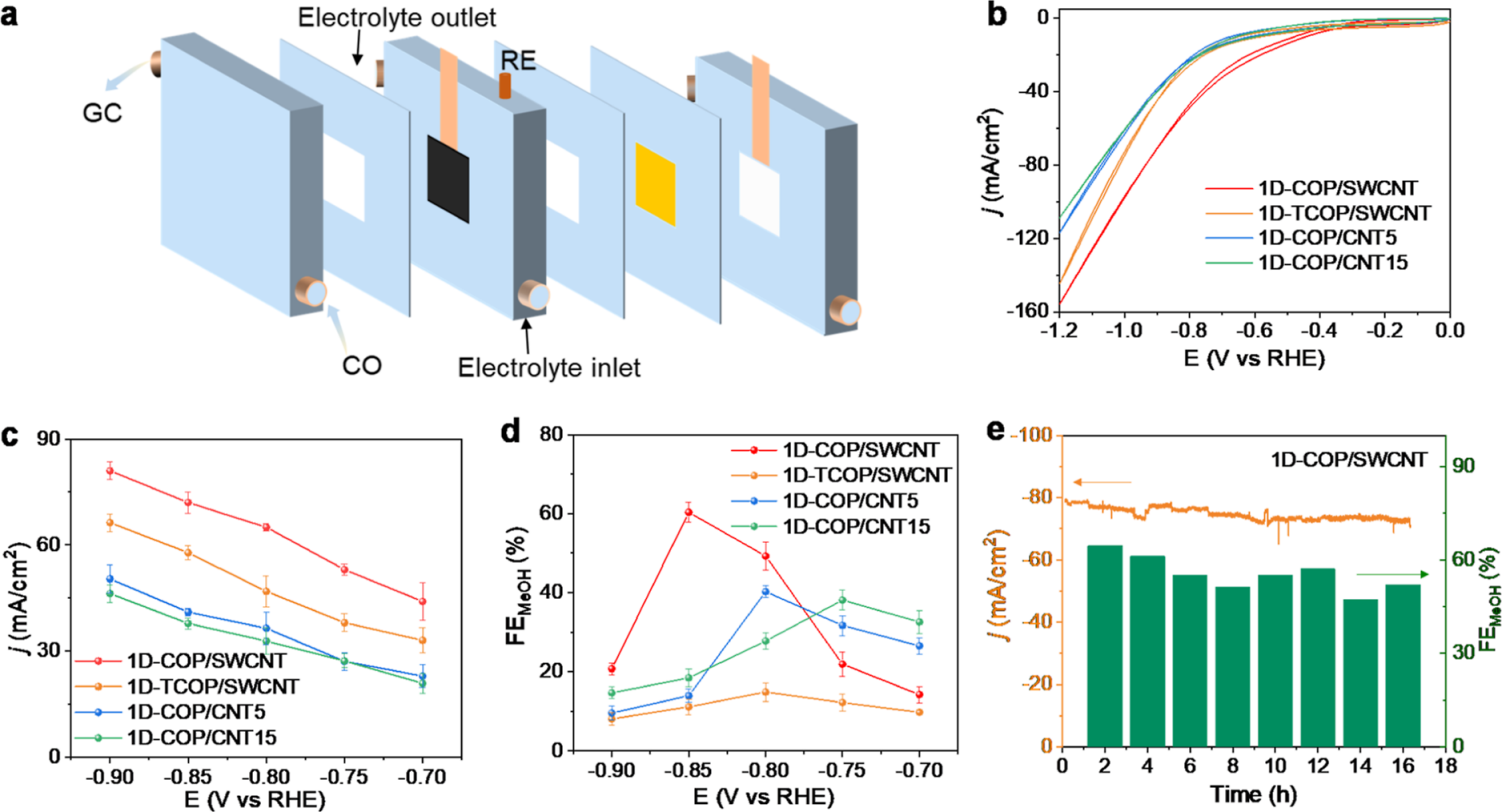
(a) Schematic illustration of the flow cell. (b) Cyclic voltammograms (CV) curves. (c) Total current density and (d) FE_MeOH_ of 1D-COP/SWCNT, 1D-TCOP/SWCNT, 1D-COP/CNT5, and 1D-COP/CNT15 with 0.1 M KOH+1.5 M KCl as the electrolyte. (e) CORR stability test of 1D-COP/SWCNT at −0.85 V versus RHE without *iR* compensation. The error bars indicate the s.d. among values from three repeated measurements.

The CORR stability of 1D-COP/SWCNT was further investigated at −0.85 V. As shown in Figure 4e, the 1D-COP/SWCNT could be operated continuously at a current density of 78 mA/cm^2^ with an average FE_MeOH_ of 58% (Figure S16). Additionally, the structure was preserved, with cobalt sites evenly distributed and no noticeable agglomeration. This is attributed to the strong covalent bonding between CoPc molecules within the COP framework (Figures S17–S21).

*In situ* FTIR measurements were performed to understand the reaction mechanism and intermediates involved in the CORR. The 1D-COP/SWCNT is shown in Figures 5a and S22. A broad peak located at 1649 cm^–1^ is attributed to the mode of the bending of the H_2_O molecules. The peak at 1919 cm^–1^, which could be attributed to the adsorption of CO on cobalt sites,^42^ gradually shifts to 1904 cm^–1^ upon when the voltage is applied from −0.5 V to −0.9 V. Improving the *CO adsorption is essential for its subsequent hydrogenation to ultimately form methanol because CoPc inherently shows moderate *CO adsorption.^48–50^ Due to the low coverage of *CHO and *OCH_3_, only the *CO intermediate can be identified. In particular, the *CO frequency signal could be observed on both 1D-COP/SWCNT and 1D-COP/CNT15 at −0.3 V, and the peak position is initially observed at 1919 cm^–1^. The intensity and the *CO area are increased due to more *CO intermediates on the Co sites. At the same time, the frequency exhibits a red-shift to a lower wavenumber when more cathodic potential is applied, which is related to the Stark effect.^51^ The *CO frequency on 1D-COP/SWCNT is lower than that on 1D-COP/CNT15, suggesting a lower *CO binding strength for 1D-COP/CNT15 (Figure 5c). A similar *CO frequency change is also found on 1D-COP/CNT15 with increasing cathodic potential from −0.5 V to −0.9 V (Figure 5b, c). However, the *CO/H_2_O area ratio of 1D-COP/SWCNT is much larger than that of 1D-COP/CNT15 (Figure 5c), indicating that the curvature of COP could affect the adsorption behavior of *CO. More adsorbed *CO on the cobalt sites could promote the reduction reaction to form methanol.^23^ We further investigated the Co-*CO bond strength under various potentials in a CO-saturated electrolyte using *in situ* Raman spectroscopy (Figure S23). Two characteristic Raman peaks at 1342 and 1505 cm^–1^ are observed across all four catalysts, corresponding to C=C and C–N vibrations of the phthalocyanine ring.^52^ A distinct *CO signal at around 1990 cm^–1^ is detected on 1D-COP/SWCNT and CNT5 but absent in other samples. This suggests that a higher degree of curvature improves *CO–Co interactions.

**Figure 5:**
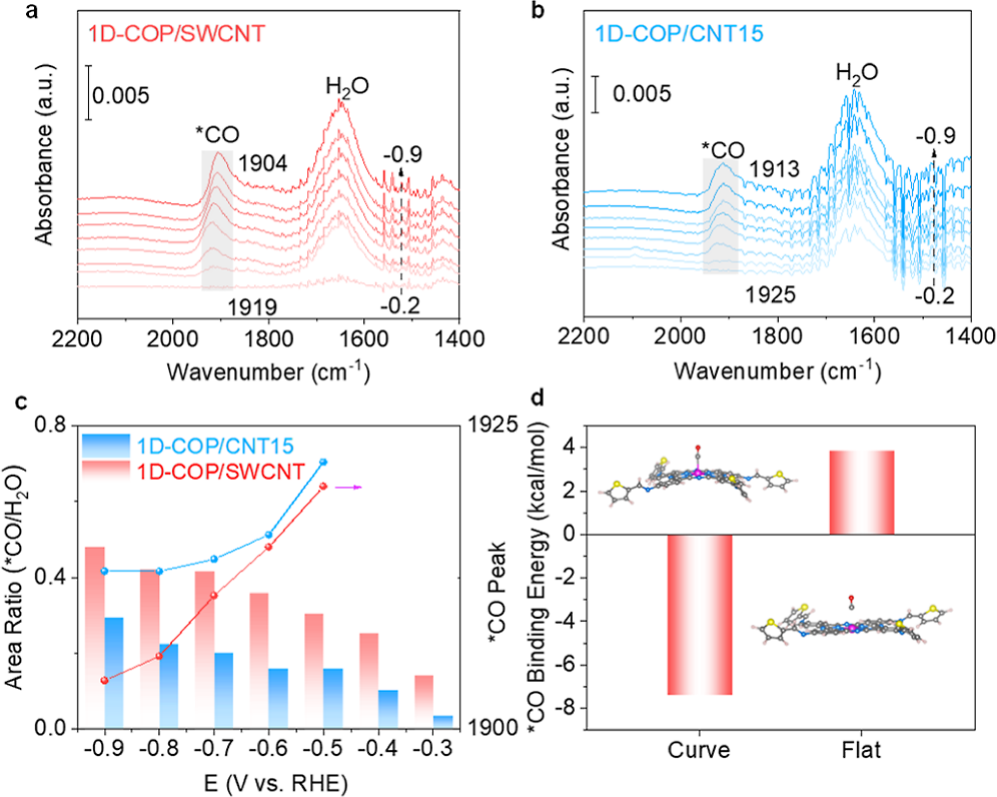
*In situ* FTIR spectra of 1D-COP/SWCNT (a) and 1D-COP/CNT15 (b) measured at −0.2 V ∼ −0.9 V vs RHE in CO-saturated 0.1 M KOH electrolytes. (c) Calculated ratio of *CO/H_2_O over Co sites of CoTAPc (bar graph) and the wavenumber shift over different potentials (dotted line). (d) The *CO binding energy of curved CoTAPc and flat CoTAPc structures. The insets in the figure show the DFT-optimized structures of curved CoTAPc-CO (top) and flat CoTAPc-CO (bottom).

To gain a deeper understanding of the CORR mechanism, DFT-optimized structures and calculations of 1D-COP/SWCNT and 1D-COP/CNT15 are listed in Figure 5d. We compare the *CO binding strength of curved and flat structures, as previous reports suggest that the stronger *CO binding strength favors the deep reduction of CO. As expected, 1D-COP/SWCNT with stronger distortion of CoPc shows more favorable *CO binding (−7.4 kcal/mol) than 1D-COP/CNT15 (3.8 kcal/mol), which is essential for the subsequent hydrogenation to form the final methanol product. The DFT results are consistent with our previous report and agree well with the experimental data in this work.

## Conclusion

In conclusion, we have successfully synthesized 1D CoPc-based COP catalysts by using the CNT as an ideal conducive support and template. We demonstrate the role of SWCNTs in inducing the distortion of local structures to tailor the CORR activity and selectivity. 1D-COP/SWCNT with stronger distortion exhibit excellent electrochemical CORR performance for methanol production. The total current density can reach 9.6 mA/cm^2^ with a good methanol FE of 70% in an H-cell, and 72 mA/cm^2^ with a FE_MeOH_ as high as 60% in a flow cell. This CORR performance exceeds other multiwalled CNT-based catalysts. The more favorable *CO intermediate absorbed on cobalt sites can be observed in *in situ* FTIR spectra, supporting that the stronger interaction with *CO over 1D-COP/SWCNT promotes the hydrogenation process and ultimately facilitates the methanol formation. Our study demonstrates that CoPc-based COP can alleviate the aggregation issue and provide a platform for inducing curvature-dependent activity of molecular catalysts. Other relevant materials such as metal organic frameworks and hydrogen-bonded organic frameworks can also leverage the support-induced strain effect for tailoring catalytic activities.

## Materials and Methods

### Materials

Single-wall carbon nanotubes (SWCNTs) and multiwall carbon nanotubes (CNT4-6, CNT10-20) were purchased from XFNANO. Cobalt tetra-amino phthalocyanine (CoTAPc, > 95%) was purchased from KaiYuLin pharmaceutical technology. Trieno­[3,2-*b*] thiophene-2,5-dicarbaldehyde (Ttp, 93%) was purchased from Bide. 1-Butanol and 1,2-dichlorobenzene were provided by J&K. Dipotassium hydrogen phosphate (K_2_HPO_4_, AR, 99%) and acetic acid were provided by Aladdin. Potassium hydroxide (KOH, 85%) was provided by Aladdin. Potassium chloride (KCl, 99.0%) was provided by Aladdin. Anhydrous ethanol (AR) was provided by LabScan; HCl (ACS, 37%) was provided by Anqua Global International, and HNO_3_ (>65%) was provided by Honeywell. Deionized water (Millipore Milli-Q grade) with a resistivity of 18.2 MΩ cm was used in all experiments.

### Pretreatment of CNTs

The carbon nanotubes were placed in a quartz dish and processed in a muffle furnace at a rate of 15 °C per minute from room temperature to 430 °C for 3 h in an air atmosphere and then cooled down naturally. They were then stirred in 6 M HCl at 85 °C for 12 h, cooled, centrifuged, washed with DI water to pH 7, and freeze-dried.

### Synthesis of 1D-COP/SWCNT

A Pyrex tube was filled with 24 mg of pretreated SWCNTs, and then a mixed 9 mL solution of n-butanol/1,2-dichlorobenzene (1:2, v/v) was added to the tube. The tube was then sonicated for 30.0 min. CoTAPc (16 mg, 0.025 mmol) and Ttp (9.8 mg, 0.05 mmol) were then added to the tube; the tube was sonicated for 30.0 min, and acetic acid (0.2 mL, 6.0 M) was added to the mixture. The tube was frozen in a liquid N_2_ bath and sealed under vacuum. After sealing, the tube was placed in an oven at 120 °C for 3 d. After it was cooled to room temperature, the resulting solid was collected by centrifugation and washed several times with acetone and methanol. Finally, the solid was freeze-dried. Other synthesis procedures were the same except that SWCNTs were replaced by multiwall CNT or the moles of CoTAPc and Ttp were increased for thick 1D-COP/SWCNT. COP was synthesized without a CNT.

### Electrochemical Measurements

The H cell electrochemical performance was performed using a customized three-compartment cell, as reported previously. A platinum foil and a Ag/AgCl leak-free reference (LF-2, Innovative Instrument Inc.) were used as counter and reference electrodes, respectively. The catalyst ink was prepared by ultrasonically dispersing 6 mg of the catalyst into 10 mL of a mixed solution of ethanol and Nafion (Nafion perfluorinated resin solution, 5 wt %) with a volume ratio of 9:1. *E* (versus RHE) = *E* (versus Ag/AgCl) + 0.23 + 0.0592 × pH). Cyclic voltammetry (CV) and liner sweep voltammetry (LSV) were performed in 0.5 M K_2_HPO_4_ solution (1.75 mL) at a scan rate of 50 mVs^–1^ after purging high-purity CO for 30 min. The pH value of the CO saturated 0.5 M K_2_HPO_4_ electrolyte was 9.26, which was measured with a pH meter (HI 2211, Hanna Instruments). Gas-phase products were quantified using an online gas chromatograph (Ruimin GC 2060, Shanghai) equipped with a methanizer, a Hayesep-D capillary column, a flame ionization detector for CO, and a thermal conductivity detector for H_2_. The CO flow rate was controlled at 2 sccm using a standard series mass flow controller (Alicat Scientific mc-50 sccm). Each run was 8 min long. The GC was calibrated with standard mix gas (Linde) and diluted with CO_2_ (Linde 99.999%). The flow cell contained a gas-diffusion layer, an anion exchange membrane (Fumasep FAA-3-PK-130), a platinum foil anode, and a leak-free Ag/AgCl electrode. The working electrodes were prepared by drop-casting 0.6 mL of catalyst ink (0.6 mg mL^–1^) onto the microporous side of the Sigmar 28 BC to achieve a total loading of 0.36 mg cm^–2^. The electrolytes, 0.1 M KOH + 1.5 M KCl, were circulated separately in the working and counter compartments using peristaltic pumps (Longer, BT100-2J) at flow rates of 1 mL min^–1^ and 10 mL min^–1^, respectively. The CO flow was maintained at 3 sccm using a mass flow controller. A lithium soft potentiostat was used to record the electrochemical responses.

### Characterization

SEM was performed on a Thermo-scientific Quattro S 15 kV. Low- and high-resolution STEM was performed using a spherical aberration-corrected STEM (JEM-ARM200F, JEOL Co. Ltd.) equipped with a cold-field emission gun and a DCOR probe Cs-corrector (CEOS GmbH) operated at 200 kV. The STEM images were obtained with an ADF detector with a convergent semiangle of 20.4 mrad and collection semiangles of 40–100 mrad. EELS acquisition was performed with a Gatan GIF Quantum ERS imaging filter equipped with a Gatan K2 Summit camera and a CCD camera with a convergent semiangle of 20.4 mrad and a collection semiangle of 111 mrad. ICP-OES measurements were performed on an Optima 8000 spectrometer. Samples were digested in hot concentrated HNO_3_ for 1 h and diluted to the desired concentrations (Table S1). XPS spectroscopy was performed on a Thermo Scientific Nexsa equipped with a monochromatic Al K radiation source (1486.6 eV; pass energy, 20.0 eV). XRD was performed on an XRD-7000 diffractometer (Rigakur) using a monochromatic Cu Kα beam (λ = 0.154 nm). Raman spectra were collected using a WITec RAMAN alpha 300R instrument with a laser wavelength of 633 nm. FT-IR spectra were collected on a PerkinElmer Spectrum 100 FT-IR spectrometer. In situ FTIR measurements were performed in a Nicolet iS50 FTIR spectrometer. The electrocatalyst was dropped onto a Au film to serve as the working electrode. A platinum wire and a Ag/AgCl electrode were used as counter and reference electrodes, respectively, in all experiments. *In situ* electrochemical attenuated total reflectance surface-enhanced infrared absorption spectroscopy experiments were performed in 0.1 M KOH electrolyte and recorded from 0 V to −0.9 V vs RHE. XAFS measurements were performed in the fluorescence mode by using a Lytle detector at beamline 01C1 of the National Synchrotron Radiation Research Center (NSRRC), Taiwan. The electron storage ring was operated at 1.5 GeV with a constant current of ∼360 mA. A Si(111) double-crystal monochromator was used to measure the photon energy. XANES analyses were performed using Athena software based on the IFEFFIT program to determine the structural environment of the cobalt atoms. The averaged X-ray absorption spectra were first normalized to the absorption edge height, and the background was removed by using the automatic background subtraction routine AUTOBK implemented in Athena software. A cobalt reference foil was used for energy calibration of the monochromator, which was applied to all spectra. The Co K-edge calibration was set to the first inflection point of the reference foil, which was set at 7709 eV for easy comparison with other work.

## Supplementary Material



## Notes

Product analysis, EXAFS fitting result, EXAFS, Raman, XRD, SEM, TEM, XPS, LSV, CV, FTIR, ^1^H NMR spectra, and *i*–*t* curves (PDF)
