# Supplementary material for: Deformed One-Dimensional Covalent Organic Polymers for Enhanced CO Electroreduction to Methanol
Source: ACS Nano. 2025 Jun 18;19(25):23370–8. doi: 10.1021/acsnano.5c06511 (PMC12755199; doi:10.1021/acsnano.5c06511)
Supplement: Supplementary file 1 [file nn5c06511_si_001.pdf]

Supporting Information

Deformed one-dimensional covalent organic polymers for enhanced CO electroreduction to methanol

Yong Liu[†], Honglei Wang[‡], Yun Song[†], Charles B. Musgrave III[ƻ], Pei Xiong[€], Jiangtong Li, Geng Li[†], Libei Huang[₸], Jianjun Su[†], Yinger Xin[†], Qiang Zhang[†], Weihua Guo[†], Mingming He[†], Tanglue Feng[†], Xing Li[†], Molly Meng-Jung Li[€], Peter A. van Aken[£], Hongguang Wang[,£], William A. Goddard III*[,ƻ], Ruquan Ye*[,†]*

[†]Department of Chemistry, State Key Laboratory of Marine Pollution, City University of Hong Kong, Hong Kong, 999077, China.

[‡]Chair Materials for Electrical Engineering and Electronics, Institute of Materials Science and Engineering and Institute of Micro and Nanotechnologies MacroNano, TU Ilmenau, Gustav-Kirchhoff-Str. 5, 98693 Ilmenau, Germany.

[ƻ]Materials and Process Simulation Center, California Institute of Technology, Pasadena, CA 91125, USA.

[€]Department of Applied Physics, Hong Kong Polytechnic University, Hong Kong 999077, China.

[₸]Division of Science, Engineering and Health Study, School of Professional Education and Executive Development, The Hong Kong Polytechnic University (PolyU SPEED), Hong Kong 999077, China.

[£]Max Planck Institute for Solid State Research, Heisenbergstr. 1, 70569 Stuttgart, Germany.

E-mail: hgwang@fkf.mpg.de; wag@caltech.edu; ruquanye@cityu.edu.hk

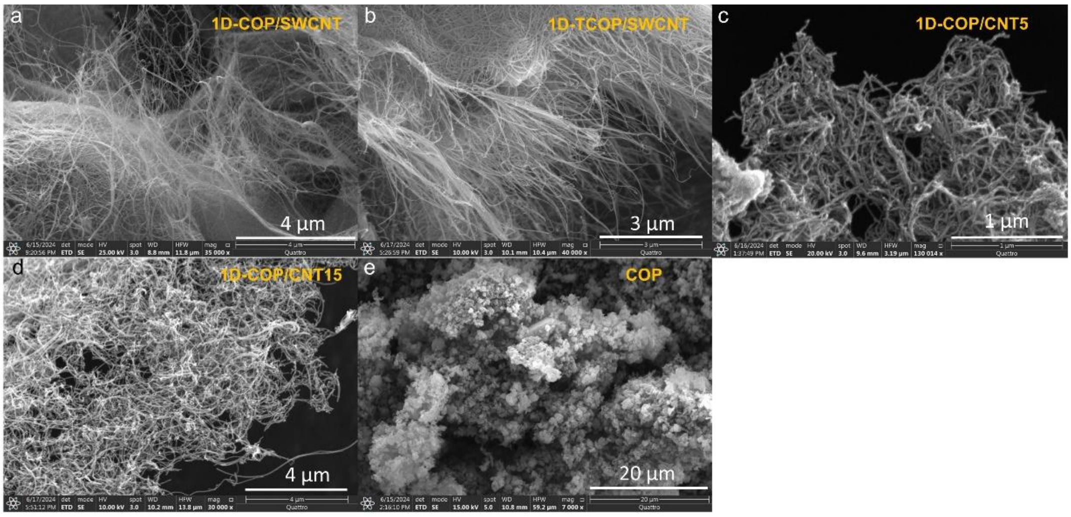

Figure S1. SEM picture of 1D-COP/SWCNT (a), 1D-TCOP/SWCNT (b), 1D-COP/CNT5 (c), 1D-COP/CNT15 (d) and COP (e).

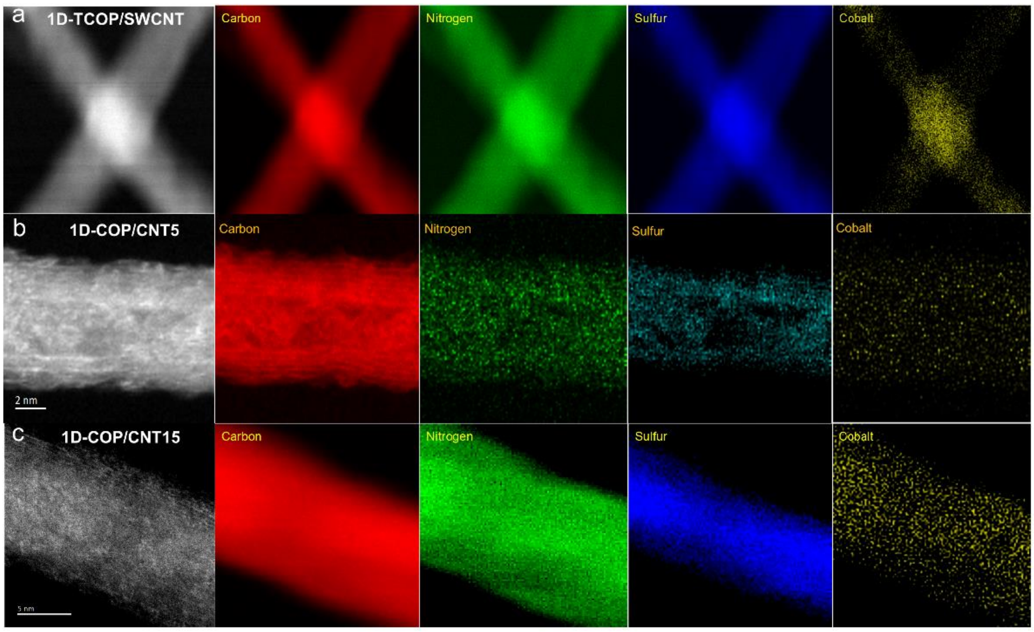

Figure S2. LAADF-STEM picture of 1D-TCOP/SWCNT (a), 1D-COP/CNT5 (b), 1D-COP/CNT15 (c) and corresponding EELS elemental maps of C (red), N (green), S (light blue), Co (yellow).

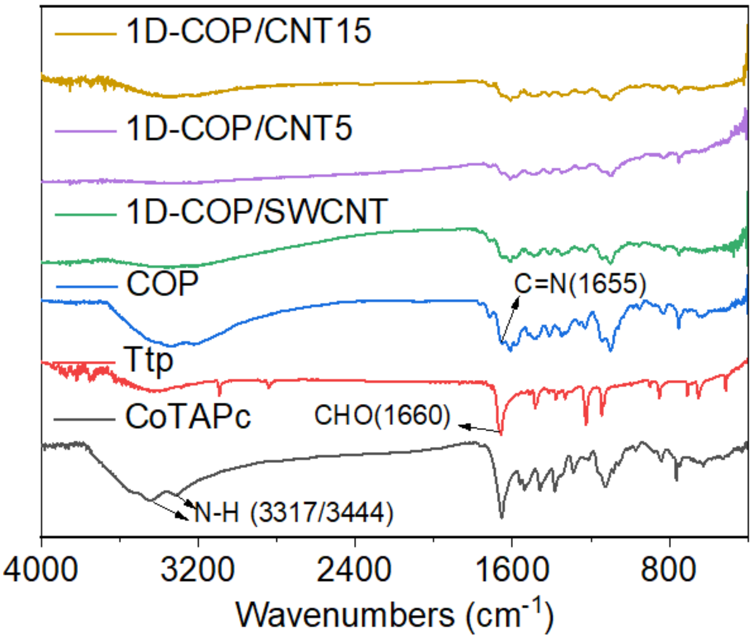

Figure S3. FTIR spectra.

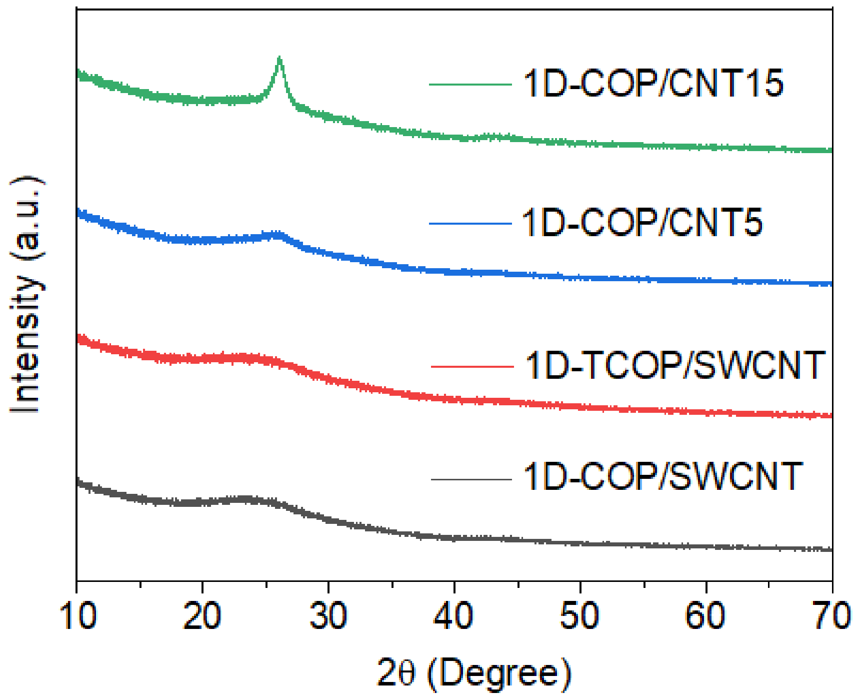

Figure S4. XRD spectra of 1D-COP/SWCNT, 1D-TCOP/SWCNT, 1D-COP/CNT5, and 1D-COP/CNT15.

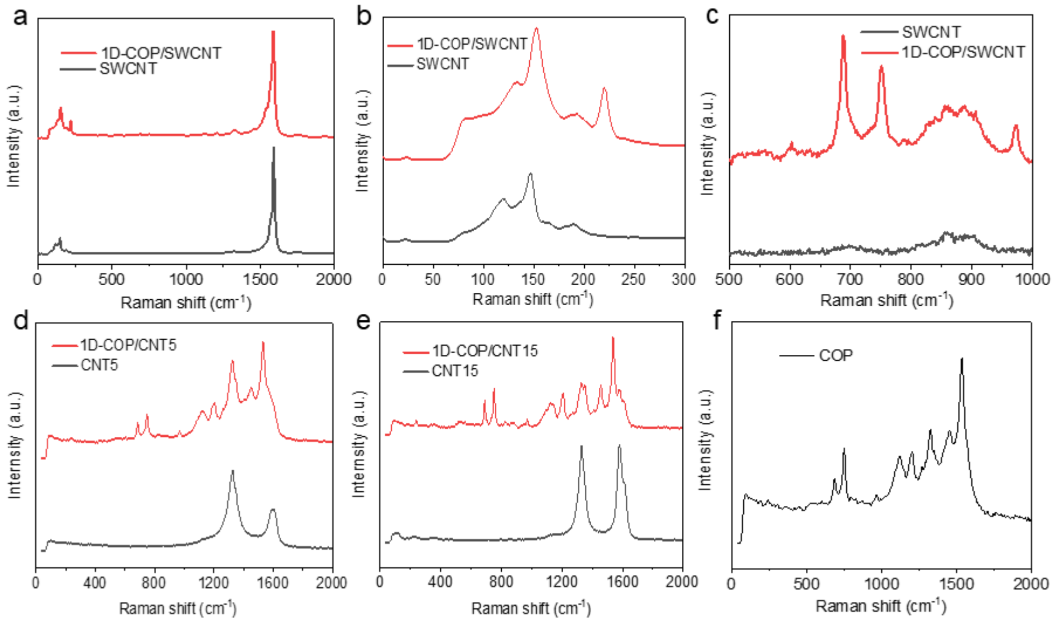

Figure S5. Raman spectra of 1D-COP/SWCNT (a-c), 1D-TCOP/CNT5 (d), 1D-COF-CNT15. (e) and COP(f).

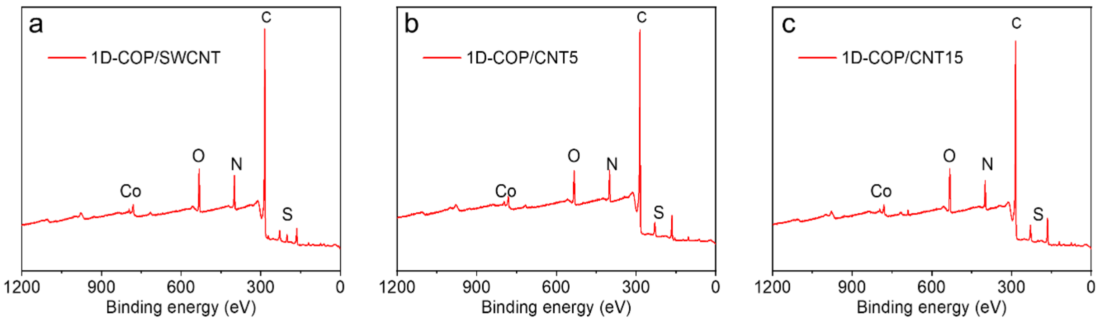

Figure S6. XPS survey of 1D-COP/SWCNT (a), 1D-COP/CNT5 (b) and 1D-COP/CNT15 (c).

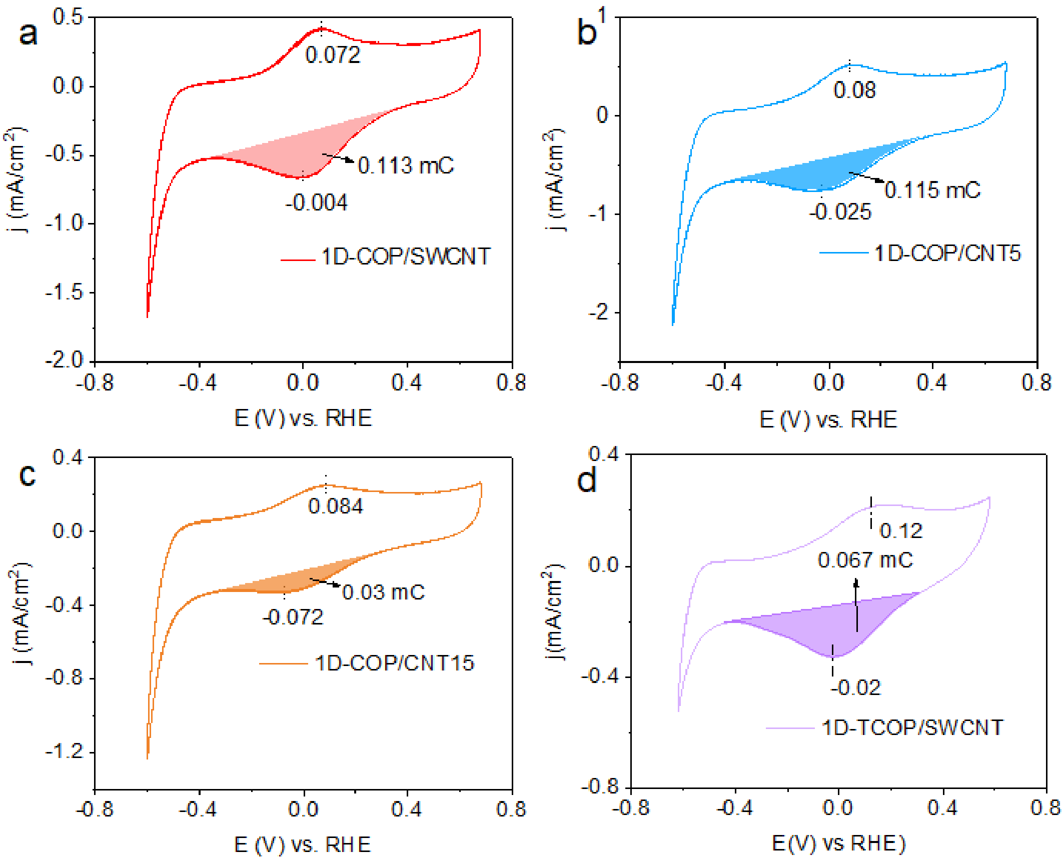

Figure S7. CV curves of 1D-COP/SWCNT (a), 1D-COP/CNT5 (b), 1D-COP/CNT15 (c) and 1D-TCOP/SWCNT in 0.1 M KOH with the scan rate of 50 mV/s.

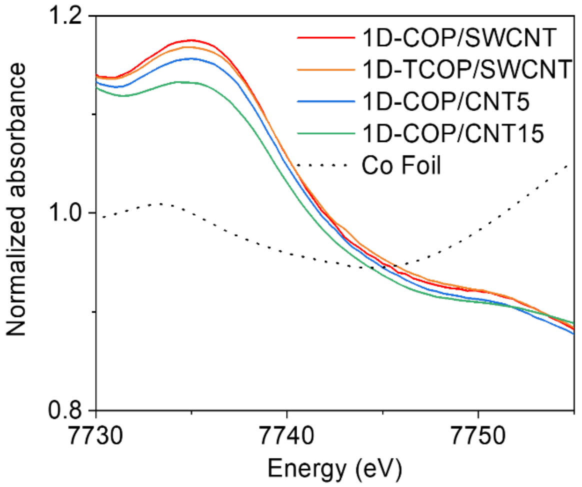

Figure S8. 1*s*-4*Px*, *Py* transition of 1D-COP/SWCNT, 1D-TCOP/SWCNT, 1D-COP/CNT5, and 1D-COP/CNT15.

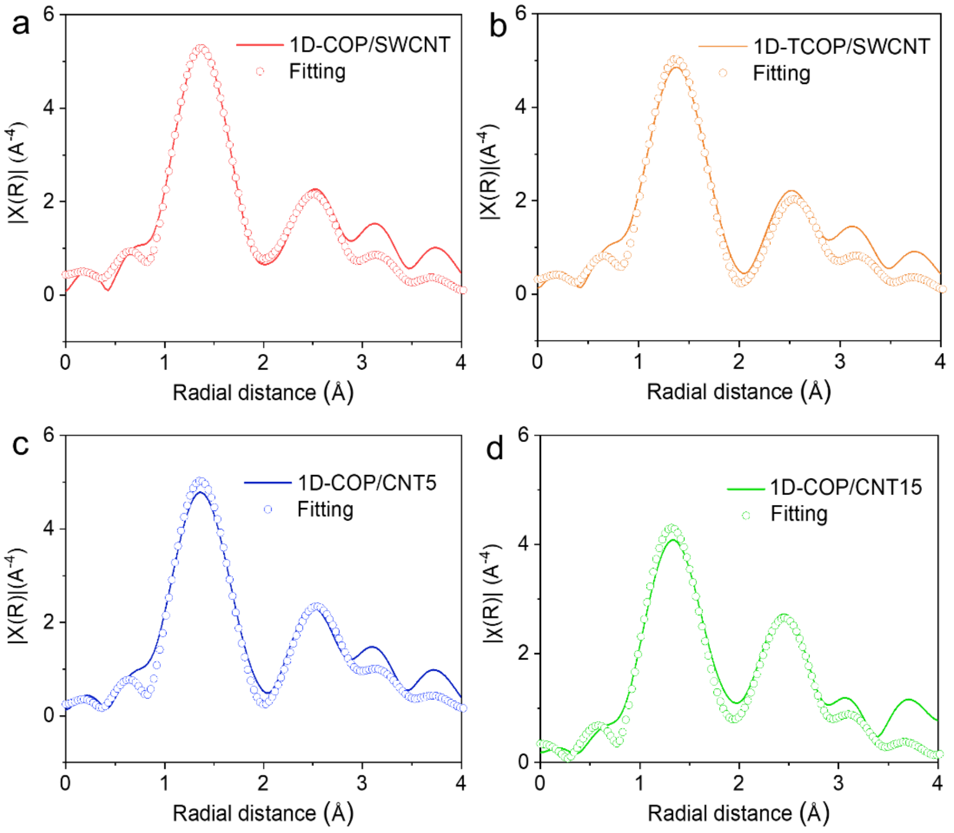

Figure S9. EXAFS Fitting in R space of 1D-COP/SWCNT (a), 1D-TCOP/SWCNT (b),1D-COP/CNT5 (c) and 1D-COP/CNT15 (d).

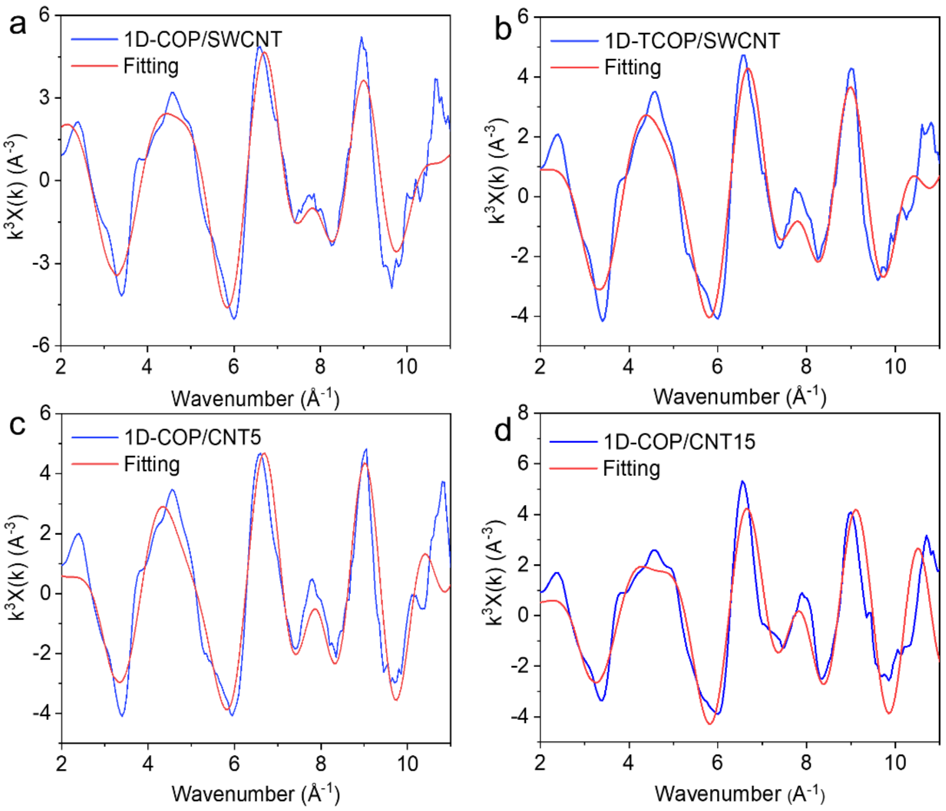

Figure S10. EXAFS Fitting in k space of 1D-COP/SWCNT (a), 1D-TCOP/SWCNT (b),1D-COP/CNT5 (c) and 1D-COP/CNT15 (d).

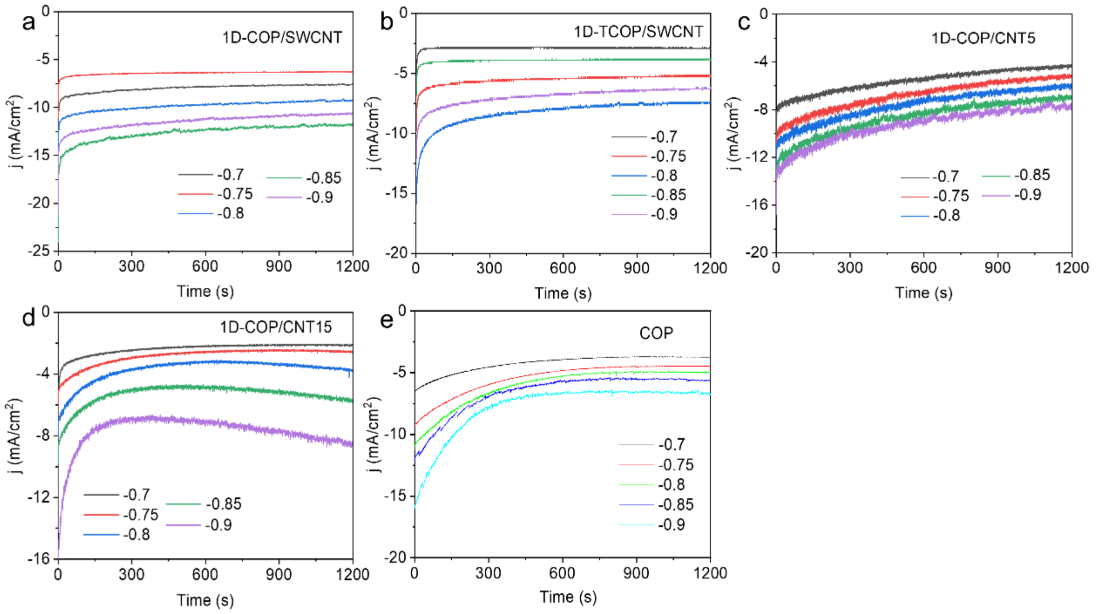

Figure S11. I-t curve of 1D-COP/SWCNT (a), 1D-TCOP/SWCNT (b), 1D-COP/CNT5 (c), 1D-COP/CNT15 (d), COP (e) in H-cell with the electrolyte (0.5M K_2HPO_4).

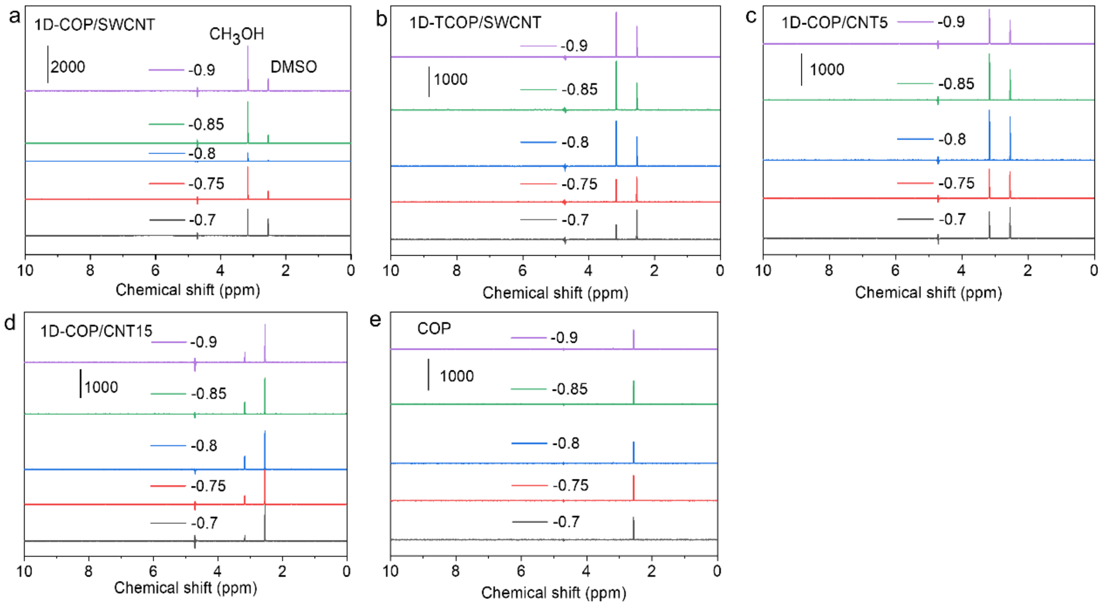

Figure S12. ^{1}H NMR spectra of 1D-COP/SWCNT (a), 1D-TCOP/SWCNT (b), 1D-COP/CNT5 (c), 1D-COP-CNT15 (d), COP (e) in H-cell.

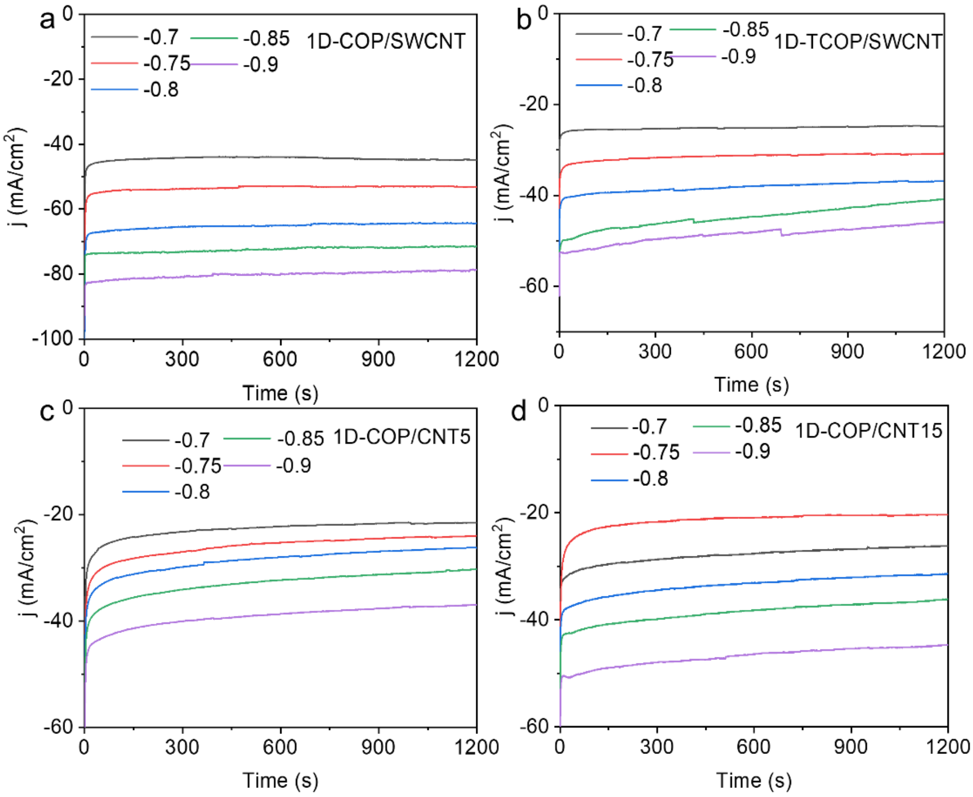

Figure S13. I-t curve of 1D-COP/SWCNT (a), 1D-TCOP/SWCNT (b), 1D-COP/CNT5 (c), 1D-COP/CNT15 (d) in flow cell with the mixed electrolytes (0.1M KOH+1.5M KCl).

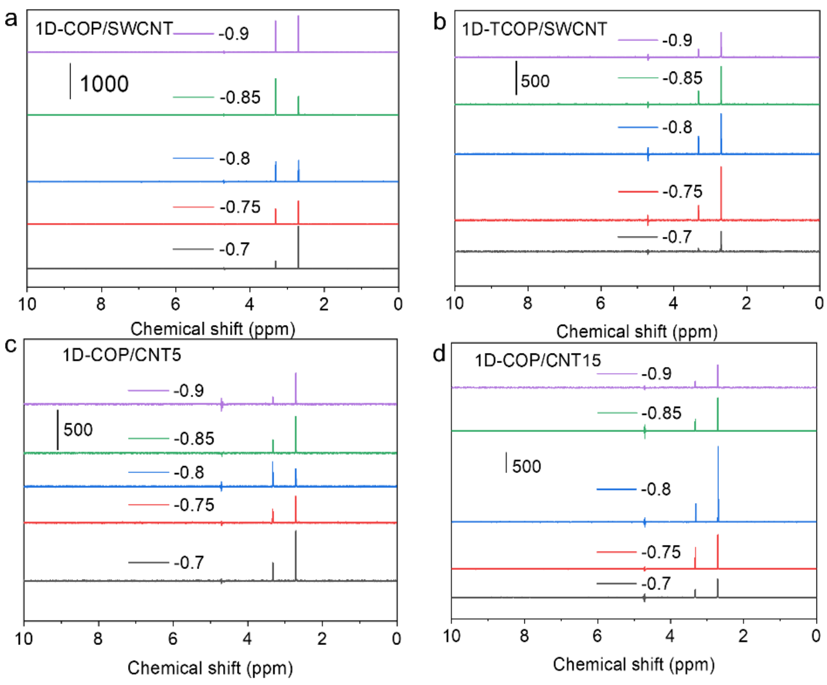

Figure S14. ^{1}H NMR spectra of 1D-COP/SWCNT (a), 1D-TCOP/SWCNT (b), 1D-COP/CNT5 (c), 1D-COP/CNT15 (d).

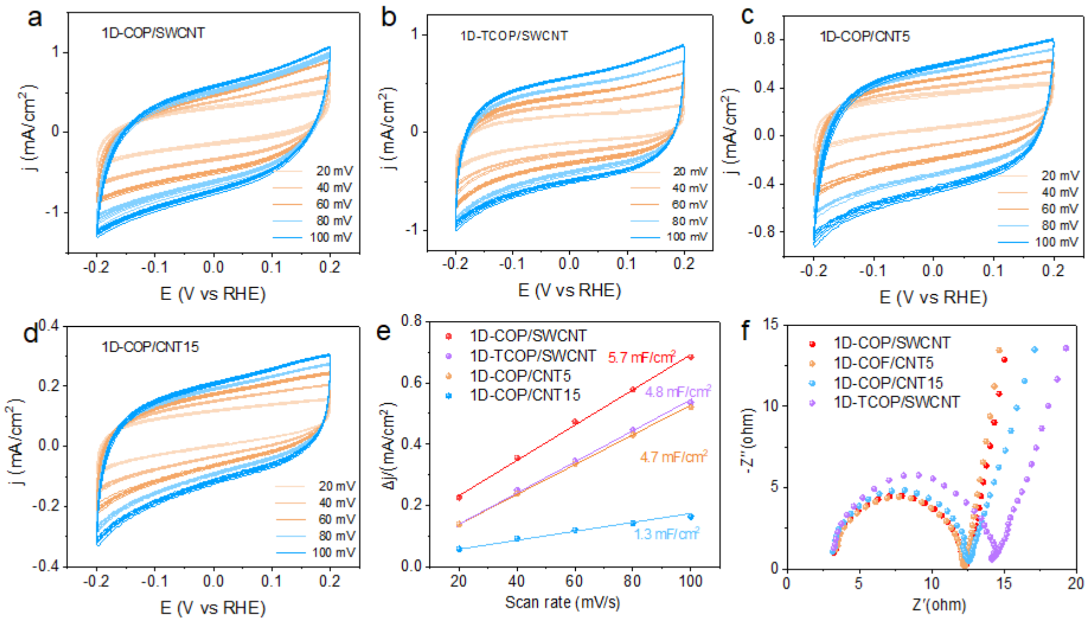

Figure S15. Effective electrochemical active surface area (ECSA) tests of (a) 1D-COP/SWCNT, (b) 1D-TCOP/SWCNT, (c) 1D-COP/CNT5 and 1D-COP/CNT15; (d) electrochemical double-layer capacity (Cdl) of four samples. (f) Nyquist plots of samples. Data was collected at an open circuit voltage in a frequency range from 100 kHz to 100 Hz at -0.25 V vs. RHE in CO-saturated 0.5 M K_2HPO_4 electrolyte.

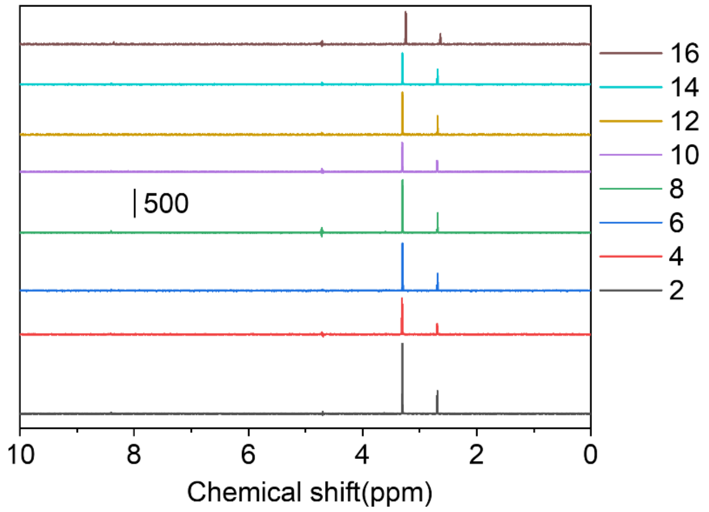

Figure S16. ^{1}H NMR spectra of 1D-COP/SWCNT in 16 h.

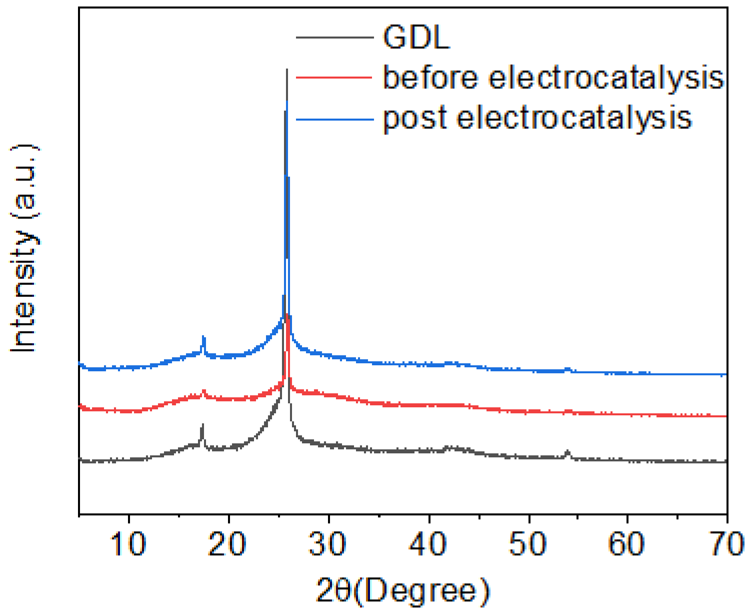

Figure S17. XRD spectra of 1D-COP/SWCNT before and post electrocatalysis.

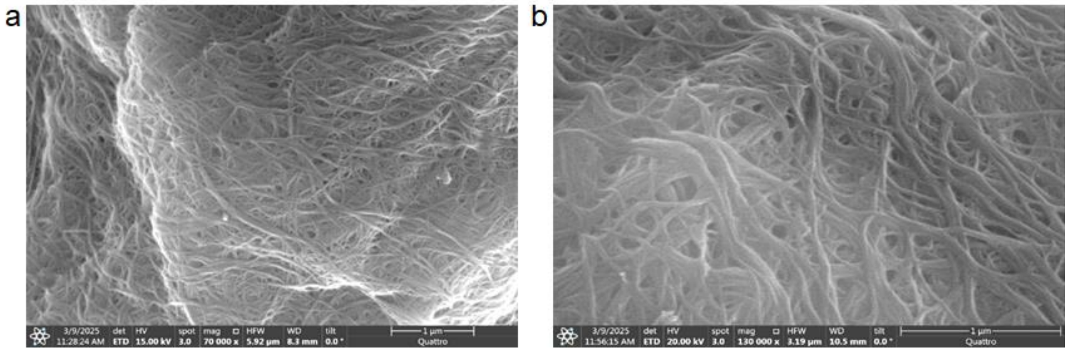

Figure S18. Raw(a) and post(b) electrocatalysis SEM of 1D-COP/SWCNT onto the GDE.

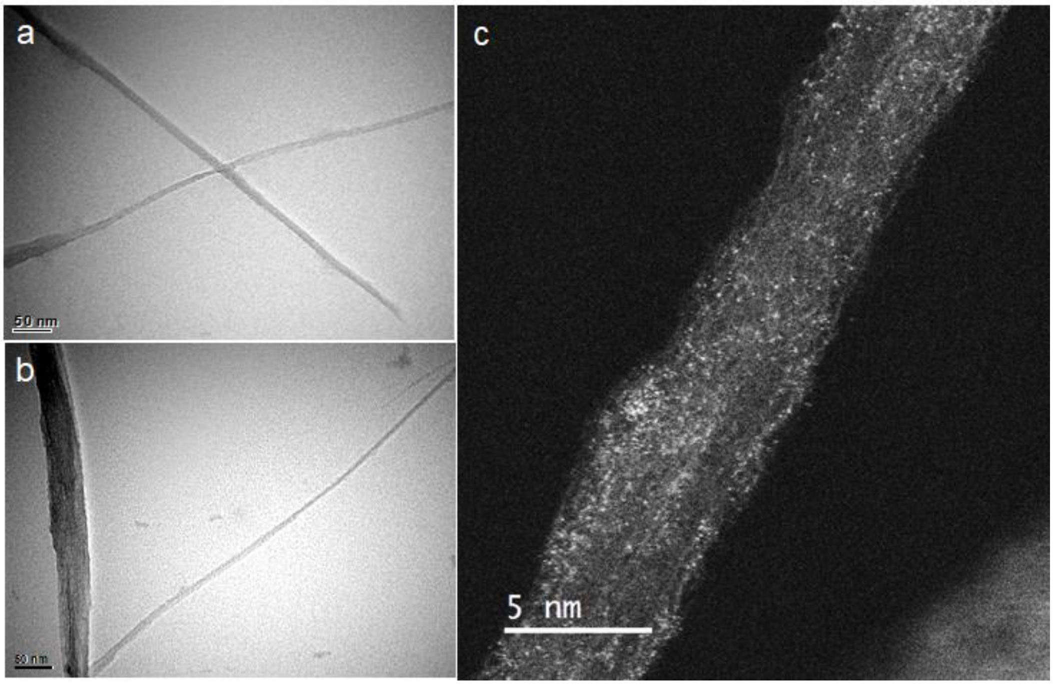

Figure S19. Raw (a) and post(b) electrocatalysis TEM and LAADF-STEM (c) of 1D-COP/SWCNT.

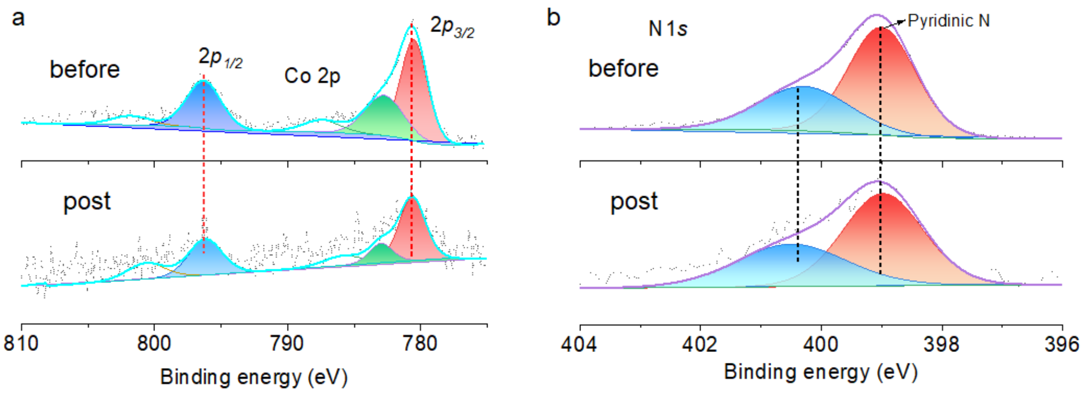

Figure S20. Co 2*p* and N 1s spectrum before and after electrocatalysis of 1D-COP/SWCNT.

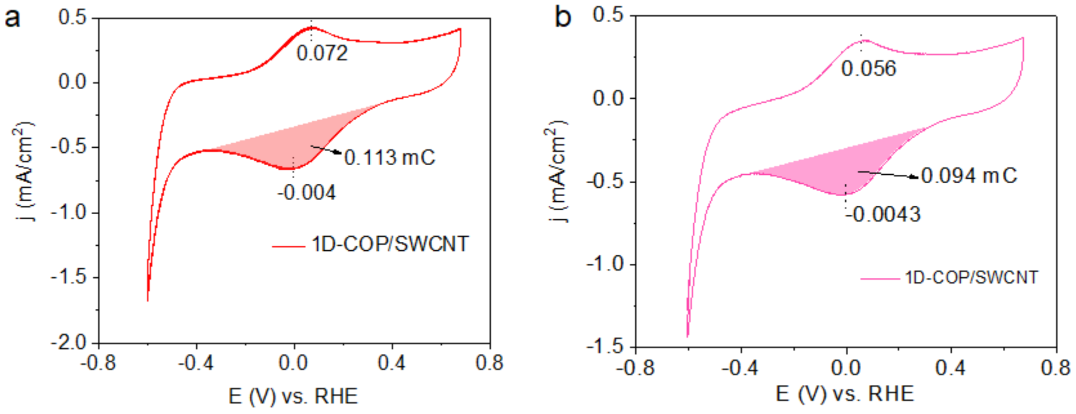

Figure S21. CV curves of 1D-COP/SWCNT before (a) and after electrocatalysis in 0.1 M KOH with the scan rate of 50 mV/s.

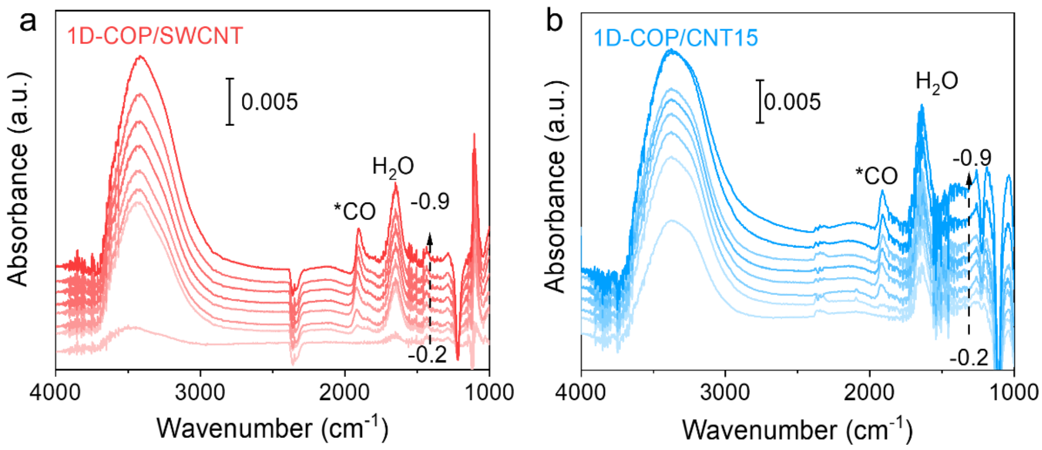

Figure S22. *In-situ* FTIR spectra recorded on 1D-COP/SWCNT (a) and 1D-COP/CNT15 (b) in CO-saturated 0.1M KOH.

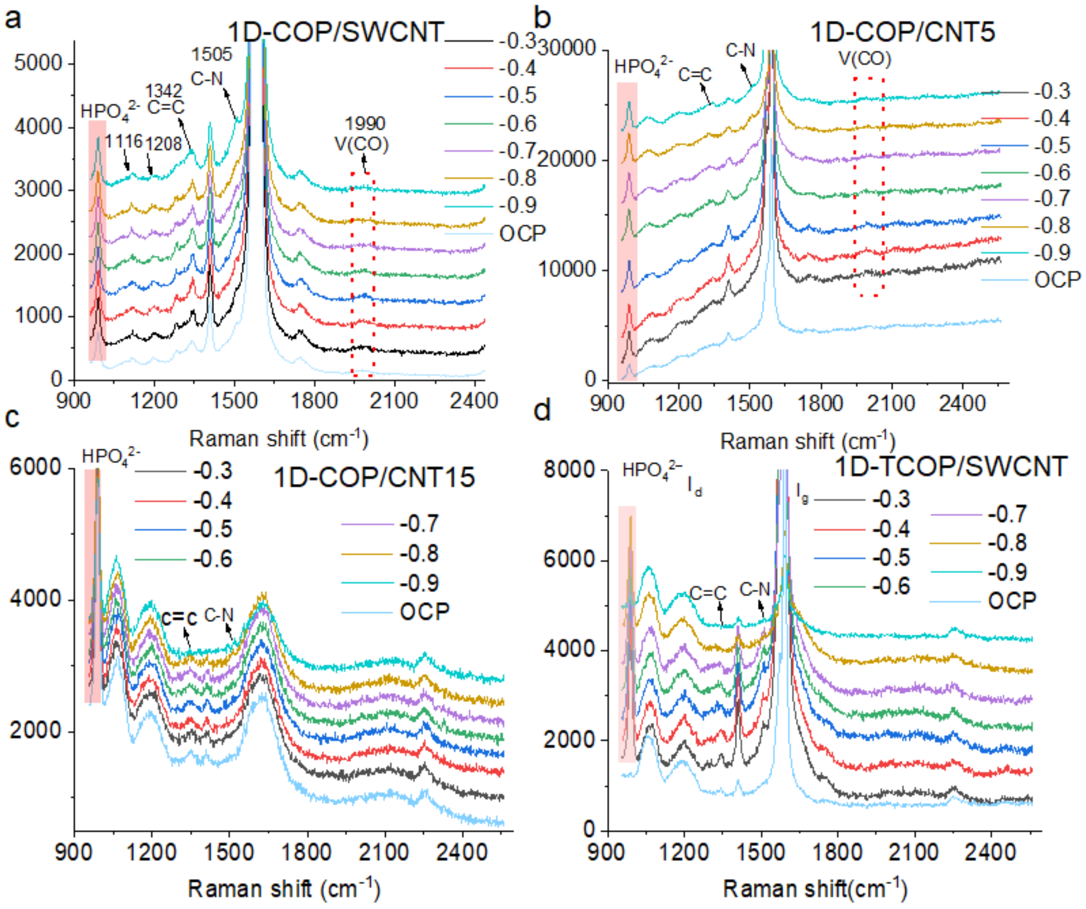

Figure S23. *In-situ* Raman spectroscopy (900-2550 cm^{-1}) of 1D-COP/SWCNT (a); 1D-COP/CNT5 (b); 1D-COP/CNT15 and 1D-TCOP/SWCNT (d) at various potentials in CO-saturated K_2HPO_4 solution.

Table S1: the ICP results of catalysts.

catalyst	Co (wt%)
1D-COP/SWCNT	1.21
1D-TCOF/SWCNT	2.53
1D-COP/CNT5	1.25
1D-COP/CNT15	1.14

Table S2: Co K-edge EXAFS fitting results, in which CN is the average coordination number, R is the distance from the absorber atom, and σ^2 the Debye-Waller factor. R-factor denotes the quality factor of the fitting, and ΔE_0 the energy shift from the absorption edge energy E_0.

Sample	Path	CN	ΔR, Å	R, Å	D-W factor (σ^2), Å^2	ΔE_0, eV	R-factor
1D-COP/SWCNT	Co-N1	**4.6 ± 0.1**	-0.02 ± 0.01	**1.89 ± 0.01**	0.006 ± 0.001	-0.3 ± 0.2	0.3%
	Co-C1	6.9 ± 0.6	-0.07 ± 0.03	2.88 ± 0.03	0.007 ± 0.001	-0.3 ± 0.3	
	Co-C2 (in CNT)	2.9 ± 0.7	-0.04 ± 0.02	3.22 ± 0.02	0.003 ± 0.002	-0.3 ± 0.3	
	Co-N2	3.1 ± 0.7	-0.05 ± 0.02	**3.30 ± 0.02**	0.009 ± 0.003	-0.3 ± 0.2	
1D-TCOP/SWCNT	Co-N1	**4.3 ± 0.2**	-0.03 ± 0.01	**1.88 ± 0.01**	0.006 ± 0.001	-2.3 ± 0.5	1.3%
	Co-C1	5.4 ± 0.6	-0.08 ± 0.03	2.86 ± 0.03	0.006 ± 0.003	-8.6 ± 0.8	
	Co-C2 (in CNT)	3.8 ± 0.7	-0.09 ± 0.02	3.18 ± 0.02	0.003 ± 0.002	-8.6 ± 0.8	
	Co-N2	2.8 ± 0.8	-0.06 ± 0.03	3.29 ± 0.03	0.006 ± 0.005	-2.3 ± 0.5	
1D-COP/CNT5	Co-N1	**4.0 ± 0.2**	-0.04 ± 0.01	**1.87 ± 0.01**	0.005 ± 0.001	-3.8 ± 0.6	1.9%
	Co-C1	5.3 ± 0.5	-0.09 ± 0.03	2.86 ± 0.03	0.004 ± 0.003	-9.6 ± 0.8	
	Co-C2 (in CNT)	4.6 ± 0.7	-0.11 ± 0.02	3.16 ± 0.02	0.003 ± 0.002	-9.6 ± 0.8	
	Co-N2	2.8 ± 0.8	-0.10 ± 0.04	3.25 ± 0.04	0.004 ± 0.004	-3.8 ± 0.6	
1D-COP/CNT15	Co-N1	**3.8 ± 0.2**	-0.05 ± 0.01	**1.86 ± 0.01**	0.005 ± 0.001	-5.8 ± 0.7	1.2%
	Co-C1	5.3 ± 0.5	-0.10 ± 0.01	2.84 ± 0.01	0.003 ± 0.001	-9.6 ± 0.8	
	Co-C2 (in CNT)	6.8 ± 0.7	-0.13 ± 0.01	3.14 ± 0.01	0.003 ± 0.002	-9.6 ± 0.8	
	Co-N2	3.8 ± 0.8	-0.05 ± 0.02	3.30 ± 0.01	0.003 ± 0.002	-5.8 ± 0.7	
